# Imaging the uptake of deuterated methionine in *Drosophila* with stimulated Raman scattering

**DOI:** 10.3389/fchem.2023.1141920

**Published:** 2023-03-29

**Authors:** Spencer J. Spratt, Takaha Mizuguchi, Hikaru Akaboshi, Hina Kosakamoto, Rina Okada, Fumiaki Obata, Yasuyuki Ozeki

**Affiliations:** ^1^ Department of Electrical Engineering and Information Systems, The University of Tokyo, Tokyo, Japan; ^2^ Laboratory for Nutritional Biology, RIKEN Center for Biosystems Dynamics Research, Kobe, Japan; ^3^ Laboratory of Molecular Cell Biology and Development, Graduate School of Biostudies, Kyoto University, Kyoto, Japan

**Keywords:** stimulated Raman scattering, methionine, deuterium, metabolic tracing, *Drosophila*, cell heterogeneity, mTOR

## Abstract

**Introduction:** Visualizing small individual biomolecules at subcellular resolution in live cells and tissues can provide valuable insights into metabolic activity in heterogeneous cells, but is challenging.

**Methods:** Here, we used stimulated Raman scattering (SRS) microscopy to image deuterated methionine (d-Met) incorporated into *Drosophila* tissues *in vivo*.

**Results**: Our results demonstrate that SRS can detect a range of previously uncharacterized cell-to-cell differences in d-Met distribution within a tissue at the subcellular level.

**Discussion**: These results demonstrate the potential of SRS microscopy for metabolic imaging of less abundant but important amino acids such as methionine in tissue.

## 1 Introduction

Small biomolecules, such as amino acids, are essential for life and play a crucial role in controlling cell fate through their participation in metabolic reactions. The metabolic profile of an individual can provide valuable information about their physiological status. However, while our understanding of the molecular details of these processes has improved, we still lack knowledge of their finer spatial details, which are important for understanding cell-to-cell differences. In cancer, such differences can lead to differential sensitivity to treatment and the emergence of resistant cells (Dagogo-Jack and Shaw, 2018). To better understand cellular heterogeneity and its implications for disease, it is necessary to visualize small biomolecules at subcellular resolution in live cells and tissue. However, this presents significant technical challenges.

Existing technologies used for medical bioimaging and metabolic profiling have limitations. Positron emission topography with magnetic resonance imaging (PET-MRI) (Castellano et al., 2021; Overcast et al., 2021) is applicable to *in vivo* imaging, but does not have the ability to resolve subcellular details. Mass spectrometry techniques (Hu et al., 2020; Goncalves et al., 2022) provide detailed molecular information and can include spatial resolution (Goncalves et al., 2022), but is destructive to cells and tissue, making it non-permissive for *in situ* testing. Click chemistry (Bird et al., 2021; Scinto et al., 2021) has more recently been used to bypass the cumbersome nature of the traditional probes in high resolution fluorescence microscopy, but these probes may not be non-invasive (Scinto et al., 2021; Spratt et al., 2022) and may not allow for the use of live cells. Additionally, most of these technologies have limited capabilities for long-term imaging.

Stimulated Raman scattering (SRS) microscopy is an imaging technique that can visualize the vibrational resonances of small biomolecules at subcellular resolution (Freudiger et al., 2008; Nandakumar et al., 2009; Ozeki et al., 2009; Ozeki et al., 2012). Its lower energy for excitation makes it well suited for use in fragile cell and tissue environments, and it has been applied in a variety of biological contexts (Cheng and Xie, 2015; Zhang and Cheng, 2018; Hill and Fu, 2019; Hu et al., 2019; Ozeki, 2020; Cheng et al., 2021). In principle, SRS can detect any molecule without the need for labeling, as each molecule has a unique spectral signature based on its bond characteristics. However, it has been difficult to specifically visualize single species among all the other molecules present. Nitriles and alkynes, which contain triple bonds (Shen et al., 2019) and have been used to label analogues (Wei et al., 2014; Wei et al., 2016), produce relatively strong signals in a region of the spectrum where most endogenous biomolecules do not. However, their submolecular differences do not allow them to fully mimic their natural counterparts, and this limits their use for long term imaging. Deuterium, or heavy hydrogen, consisting of an additional neutron, has shown promise as a submolecular label for imaging (van Manen et al., 2008). It has been used to label abundant amino acids such as leucine (Wei et al., 2013; Wei et al., 2015), as well as multiple amino acids (Wei et al., 2013; Wei et al., 2015; Wei et al., 2016). In recent work, deuterated water and the model organism *Drosophila* have further demonstrated the non-invasive nature of deuterium for systemic labelling *in vivo* and long-term imaging, highlighting its potential as a universal metabolic probe (Shi et al., 2018; Li et al., 2021; Li et al., 2022a; Li et al., 2022b; Fung et al., 2022). However, deuterium’s weaker vibrational resonance can limit the detectable signal, making it less suitable for labeling less abundant single species of biomolecules such as methionine (Met). Met is an essential amino acid and plays a crucial role in cell fate. Its metabolic product, S-adenosyl methionine (SAM), regulates stem cell division and differentiation (Shiraki et al., 2014; Obata et al., 2018), and Met is essential for cancer cell division (Hoffman and Erbe, 1976; Kaiser, 2020). In our recent work, we used deuterated methionine (d_8_-Met) and demonstrated the ability to detect the CD stretching peak of d_8_-Met by careful subtraction of background signal (Spratt et al., 2022).

**FIGURE 1:**
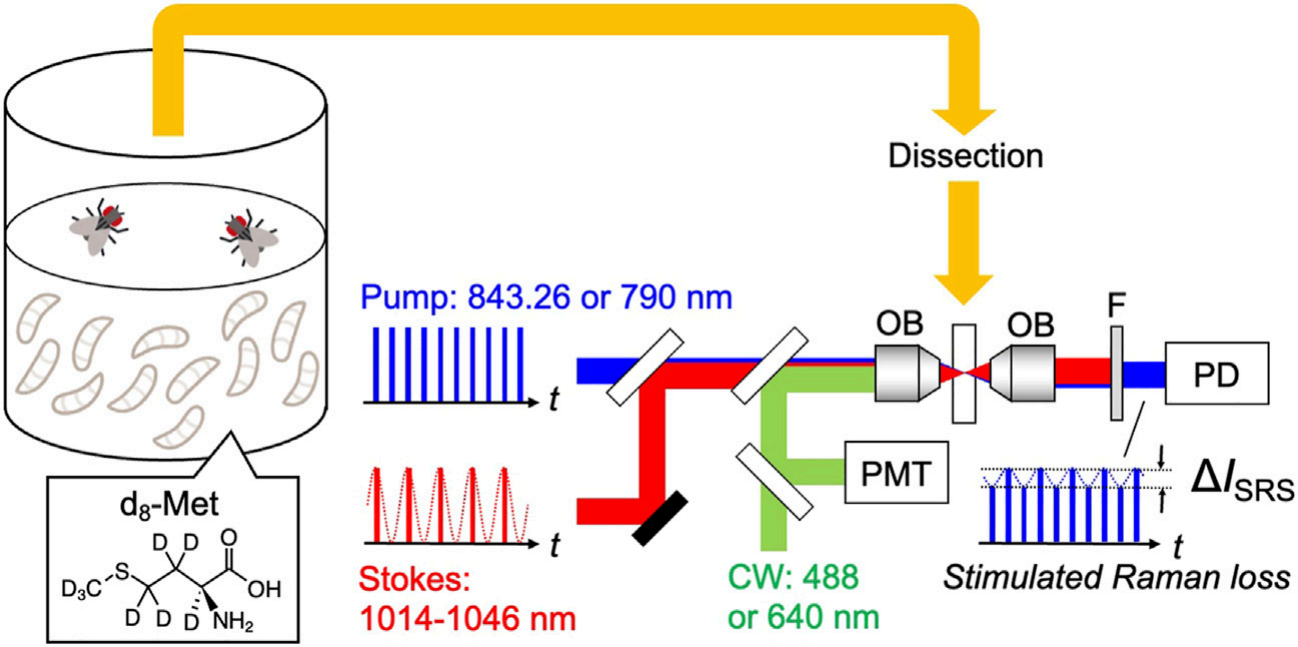
Schematic of the experiment: SRS and fluorescence imaging of tissue, dissected from *Drosophila* raised on holidic medium containing deuterated methionine (d_8_-Met). OB: objective lens, F: short-pass filter, PD: photodiode, PMT: photomultiplier tube.

Here, we expand upon this work and demonstrate SRS imaging to visualize d_8_-Met, in multiple dissected *Drosophila* tissues, incorporated systemically *in vivo*. By using our integrated SRS and fluorescence microscope (Figure 1), we were able to observe specific cells and investigate the heterogeneous nature of cells in tissue at subcellular resolution, observing cell-to-cell differences in d_8_-Met uptake and distribution. Here, we also see differences comparing tissue from *Drosophila* after genetic manipulation. We anticipate that d_8_-Met and other deuterated single species of small biomolecules have the potential to be useful for investigating metabolic processes at subcellular resolution and for understanding cell heterogeneity and cell fate.

## 2 Materials and methods

### 2.1 *Drosophila* stocks and husbandry

Flies were reared on a standard diet containing 4% cornmeal, 6% yeast, 6% glucose, and 0.8% agar with propionic acid and nipagin. The *hsFLP*, *UAS-mCD8-GFP*; *act5C-FRTstopFRT-GAL4*, *UAS-GFP* with *UAS-TorTED* genotype was used to generate marked cells and clones for imaging the fat body. The *esg-GAL4, UAS-GFP*, *tub-Gal80* ^*t*^ genotype (Obata et al., 2018) was used to mark stem cells and progenitor cells in the adult gut. The Canton S strain was used as the normal control.

### 2.2 Sample preparation for SRS imaging of *Drosophila* tissue

Flies were transferred to a modified version of holidic medium (Kosakamoto et al., 2022) supplemented with 3.82 mM d_8_-Met (Cambridge Isotope Laboratories: DLM-6797–0.1) instead of Met, for up to 48 h prior to dissection unless otherwise stated. Third instar larvae or one-week-old adult *Drosophila* were placed in phosphate buffered saline (PBS) (4°C) and dissected using forceps (Dumont, Inox, #5) under a dissecting microscope. n = 3 or greater and carried out on different days using at least two different populations. Tissues were mounted on glass coverslips (Matsunami: (24 mm × 50 mm, 0.13–0.17 mm); (round, 12 mm, 0.13–0.17 mm)) with SecureSeal imaging spacers (Grace Bio-Labs, 654008), in PBS, PBS with 4% paraformaldehyde (adult gut only), or PBS with LysoTracker Deep Red (Invitrogen, L12492), 50 μM.

### 2.3 SRS and fluorescence imaging

The integrated SRS and fluorescence imaging system used in this study is described previously (Shou et al., 2021; Spratt et al., 2022). The central wavelength of the pump pulses is fixed at 843.26 nm or 790 nm, with a spectral width of 0.15 nm for imaging the silent (2,000–2,300 cm^−1^) or CH stretching (2,800–3,100 cm^−1^) regions, respectively. The light is focused on the sample by an objective lens (Olympus, 60×, N. A. = 1.2 or Olympus, 25×, N. A. = 1.05) and the transmitted light is collected by another objective of the same type. After confirming the position of the spectral peak for d_8_-Met, measurements were taken for four spectral points each with 1,500 frames: 2,107, 2,127, 2,130, and 2,150 cm^−1^. SRS signal of d_8_-Met was obtained as $I=I_{2}+I_{3}-\left(I_{1}+I_{4}\right)$ , where $I_{i}$ corresponds to the SRS signal of the i ^th^ spectral image (Spratt et al., 2022). Lipid images with CH_2_ stretching mode were taken at 2,853 cm^−1^. Fluorescence excitation/emission was set at 488/510 nm for green fluorescent protein or 640/668 nm for LysoTracker Deep Red.

### 2.4 Data processing and image generation

SRS and fluorescence data were acquired with LabVIEW-FPGA software and processed using ImageJ to generate tissue images. For further details, please refer to (Spratt et al., 2022).

## 3 Results

### 3.1 SRS imaging of d_8_-Met in *Drosophila* tissues

**FIGURE 2:**
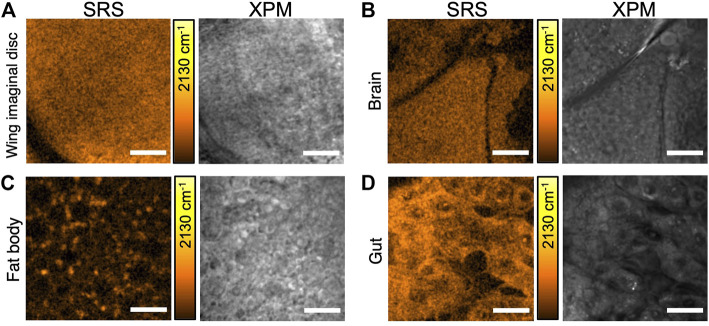
SRS imaging of d_8_-Met uptake in various *Drosophila* tissues. **(A)** larval wing imaginal disc, **(B)** larval brain, **(C)** larval fat body, and **(D)** adult gut. SRS images are shown (left side) with the corresponding background due to cross-phase modulation (XPM) (right side). The color bars corresponding to the d_8_-Met concentration shows maximum values of **(A)** 65 mM, **(B)** 40 mM, **(C)** 51 mM and **(D)** 37 mM. Scale bars 20 μm **(A, D)**, 25 μm **(B, C)**.

To evaluate the *in vivo* incorporation of d_8_-Met in whole organisms, we raised *Drosophila* larvae on a holidic medium containing d_8_-Met and used SRS imaging to detect d_8_-Met signal in various tissues after dissection. In Figure 2, we compare d_8_-Met uptake in the *Drosophila* larval wing imaginal disc, larval brain, larval fat body, and adult gut. The subcellular characteristics of the relatively small wing disc cells and larval brain cells are not easily distinguished in the images (Figures 2A,B), and the homogenous distribution of high d_8_-Met signal may be due to the high division rate of these tissues at this stage (Tripathi and Irvine, 2022). The distribution of d_8_-Met in the relatively large fat body cells appears to show localized signal at specific loci or puncta (Figure 2C). These puncta appear to be located near subcellular structures resembling lipid droplets (LDs), which are commonly present in the fat body. The SRS image of gut tissue from an adult *Drosophila* kept in the same vial for 30 days (Figure 2D) shows a high level of signal, supporting the potential of d_8_-Met for long-term incorporation *in vivo*. These results support the use of SRS imaging with a deuterated probe to distinguish cellular features at subcellular resolution, depending on the cell type and developmental stage.

### 3.2 SRS and fluorescence imaging of cell-type dependent uptake of d_8_-Met

Many tissues consist of at least two cell types at some stage during their development, including stem cells and cells that have undergone determination to differentiate into a specific cell type with specific functions. A good example of this complexity is the *Drosophila* midgut, which consists of at least five cell types (Obata et al., 2018; Hung et al., 2020; Guo et al., 2022): intestinal stem cells (ISCs), enteroblasts (EBs), enteroendocrine progenitors (EEPs), enterocytes (ECs) and enteroendocrine cells (EEs). ISCs can self-renew or be determined/specified to differentiate into ECs or EEs *via* committed EBs or EEPs progenitor cells, respectively. ECs and EEs, have specialized functions such as gut enzyme secretion and hormone secretion, respectively (Guo et al., 2022). This arrangement continues throughout development from larval stages to the adult. The relatively large size of these cells makes the *Drosophila* gut a suitable model to investigate cell-to-cell differences within a tissue.

**FIGURE 3:**
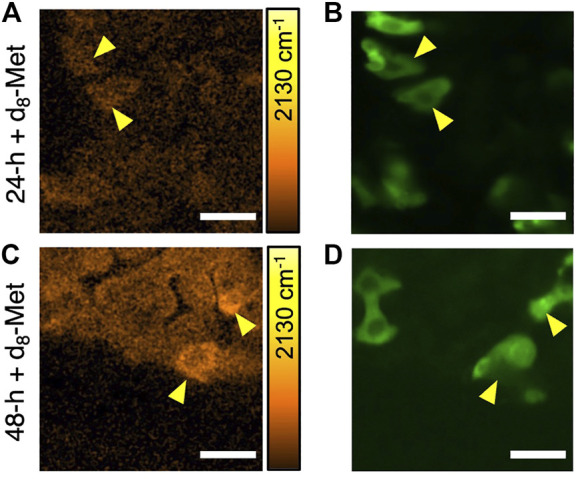
The uptake of d_8_-Met in fixed *Drosophila* adult gut cells varies by cell type. SRS images of d_8_-Met uptake (orange) after feeding on d_8_-Met food for 24 h **(A)** and 48 h **(C)**. Yellow arrowheads indicate cells with high d_8_-Met signal **(A, C)** and their corresponding *esg* directed GFP expression (green) **(B, D)**. The color bars corresponding to the d_8_-Met concentration shows maximum values of **(A)** 43 mM and **(C)** 45 mM. Scale bars, 25 μm.

To examine d_8_-Met uptake in specific cell types, we used our SRS and fluorescence microscope, and the GAL4 UAS genetic technique (Brand and Perrimon, 1993) to control and direct expression. We marked midgut progenitor cells by directing the expression of GFP under the control of the *escargot* (*esg*) gene, which is known to be expressed in cells including ISCs and EBs. In the case of the adult gut, we fixed the dissected tissue to avoid any movement during the time required for imaging. d_8_-Met uptake in *Drosophila* adult gut shows a heterogeneous distribution (Figures 3A,C). Cell-to-cell differences in d_8_-Met uptake are clearly apparent, with some of the marked GFP expressing cells showing higher d_8_-Met uptake compared to the surrounding unmarked non-GFP expressing cells (Figures 3B,D). Furthermore, not all the purported progenitor cells marked with GFP show high d_8_-Met uptake, perhaps reflecting their heterogeneous nature. However, without further experiments, at this stage we are unable to comment further about each cell’s developmental stage or whether a more detailed description of a cell’s developmental stage could potentially benefit from the highly sensitive and quantitative capabilities of SRS.

### 3.3 d_8_-Met puncta have lysosomal characteristics

**FIGURE 4:**
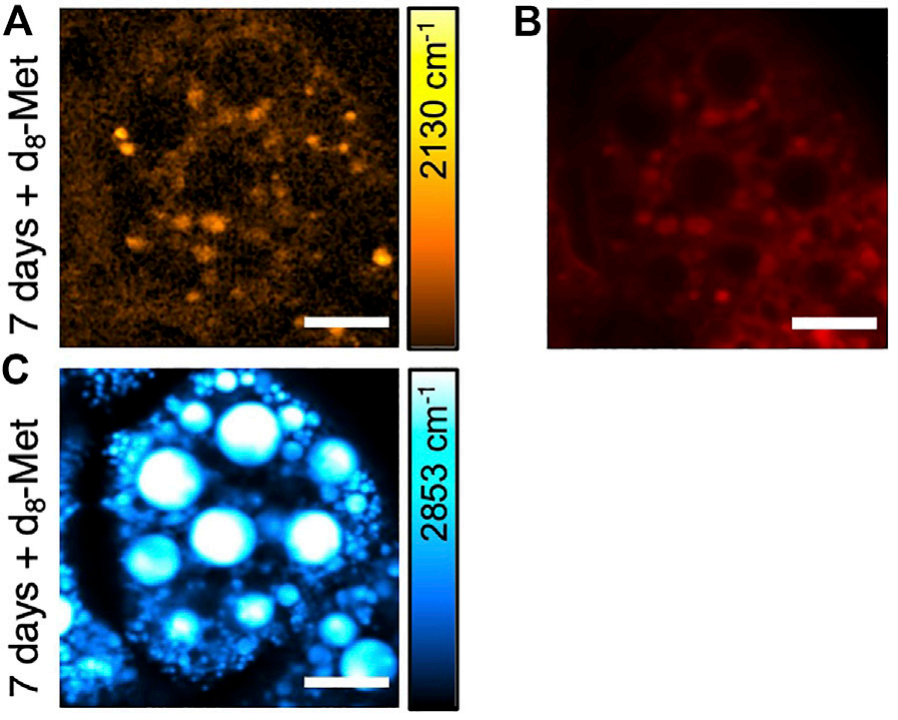
SRS and fluorescence imaging of *Drosophila* larval fat body. **(A)** d_8_-Met uptake (orange), **(B)** LysoTracker (red) and **(C)** lipid (blue). The color bar corresponding to the d_8_-Met concentration shows maximum values of **(A)** 42 mM. Scale bars, 20 μm.

To further characterize the d_8_-Met positive puncta observed in the fat body tissue, we performed SRS imaging in the CH stretching region and observed strong SRS signal from CH_2_ stretching vibrations at 2,853 cm^−1^ (Figure 4C), which is typical of -CH_2_ vibrational frequencies, suggesting high amounts of lipid typical of LDs. The d_8_-Met positive puncta observed in the fat body tissue appear to be located around LDs (Figure 4A, Figure 2C). To further investigate the puncta and LDs, we used LysoTracker—a commercially available fluorescent probe that is commonly used to track acidic organelles, previously used to mark lysosomes including those lying near or interacting with LDs in *Drosophila* larval fat body (Scott et al., 2004). Our results show that some of the d_8_-Met positive puncta colocalize with LysoTracker (Figure 4B), indicating a lower pH and a lysosomal identity. In addition, not all d_8_-Met positive puncta colocalize with LysoTracker. The putative lysosomes appear to be located around the LDs (Figures 4B,C). Taken together, these observations raise interesting questions about d_8_-Met uptake kinetics and metabolism in the fat body, and whether lysosomes and additional organelles might play a role.

### 3.4 Genetic manipulation of mTOR signaling *in vivo* can control d_8_-Met uptake

The mechanistic target of rapamycin (mTOR), a kinase that is a central regulator of cell metabolism (Simcox and Lamming, 2022), integrates signals from multiple sources to control cell fate. Deactivation of mTOR signaling can decelerate anabolic processes such as protein synthesis and lipid synthesis, and accelerate catabolic processes such as lysosome biogenesis and autophagy. Therefore, mTOR signaling is a suitable model to study the relationship between d_8_-Met uptake and the lysosome, as well as subcellular organelle biology in general.

**FIGURE 5:**
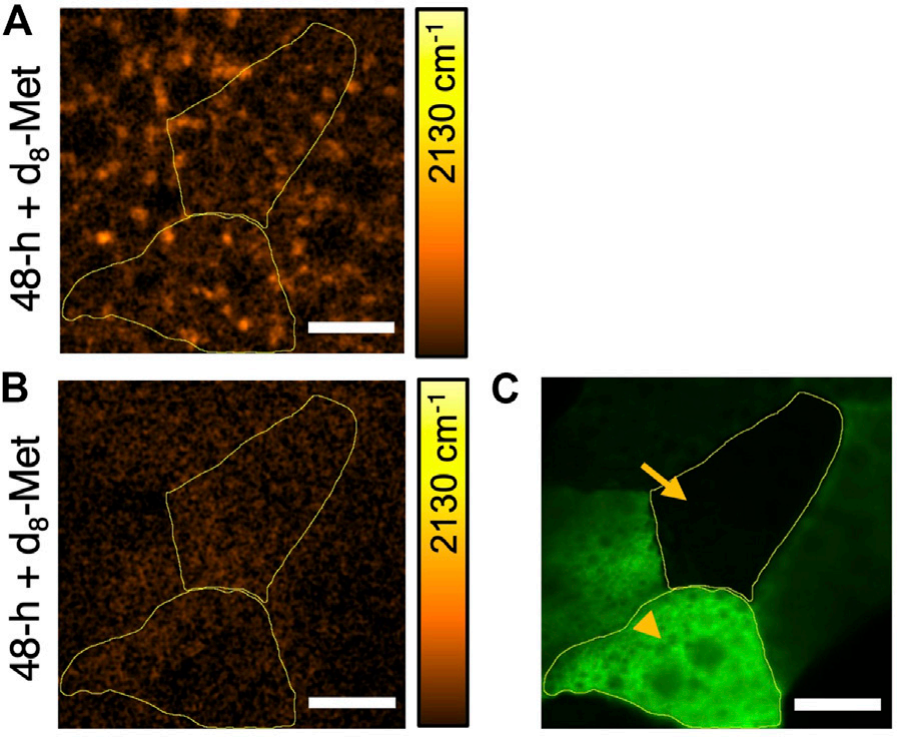
The uptake of d_8_-Met in the *Drosophila* larval fat body can vary according to genotype. d_8_-Met uptake (orange) in control **(A)** and *TorTED* expressing fat body tissue **(B)**. The flip-out technique has been used to direct *TorTED* expression in GFP marked cells (green) **(C)** and demarcated areas (yellow boundaries) correspond to regions of interest (ROI), ROI1 (arrowhead) and ROI2 (arrow). Note that **(B)** and **(C)** corresponds to the same tissue and same field of view. The average d_8_-Met concentrations in ROI1 and ROI2 are (A) 3.68 mM and 3.34 mM, (B) 1.97 mM and 2.48 mM, respectively. The color bars corresponding to the d_8_-Met concentration shows a maximum value of 51 mM, Scale bars, 20 μm.

Using the flip-out technique in *Drosophila* (Golic and Lindquist, 1989), which offers powerful genetic techniques to control biological processes *in vivo*, we directed the expression of *TorTED* (Hennig and Neufeld, 2002), a dominant negative form of mTOR that interferes and inhibits mTOR signaling in specific cells marked with GFP (Figure 5C). Remarkably, d_8_-Met uptake appears to be significantly reduced in fat body tissue with cells that have manipulated mTOR signaling (Figure 5B), suggesting that methionine uptake in *Drosophila* larval fat body is regulated by mTOR signaling. However, we do not currently understand why the lack of puncta appears to be global and does not correspond only to the pattern of GFP expression marking ectopic *TorTED* expressing cells (Figures 5B,C). One possibility is that, unlike the membrane tagged GFP, *TorTED* or a metabolic product of mTOR signaling disruption may cross membranes to affect adjacent cells. An alternative explanation might involve some other aspect of biology, perhaps related to the fat body’s important role in monitoring global homeostasis and the organism’s need for fast joint control of immediate metabolic demands.

## 4 Discussion

In this work, we performed SRS imaging on dissected *Drosophila* tissue to detect the *in vivo* systemic incorporation of d_8_-Met. We demonstrate that SRS imaging is capable of detecting cell heterogeneity within live tissue and of providing detailed information about the distribution of d_8_-Met at subcellular resolution. These findings add to the growing evidence showing that SRS imaging of small deuterated biomolecules is a highly versatile and sensitive method for long-term analysis of metabolism, with the potential to track many aspects of metabolism from uptake and distribution to downstream molecular fate (Wei et al., 2013; Wei et al., 2015; Wei et al., 2016; Shi et al., 2018; Li et al., 2021; Li et al., 2022a; Li et al., 2022b; Fung et al., 2022). The high sensitivity and quantification capabilities of SRS imaging make it a useful tool for metabolic profiling with subcellular spatial resolution, and this will be useful for studying metabolism and cellular heterogeneity in a range of biological processes, including protein metabolism in development and diseases such as cancer. This technique could also be extended to other amino acids and small biomolecules, providing insights into the relationships between nutrition, metabolism, cell heterogeneity, cell fate and physiological status. Additionally, the ability to study specific biomolecules in live cells and tissues at subcellular resolution could be used for target identification in the search for therapeutics.

It is known that SAM is a key regulator of epigenetic control during normal development and disease (Moore et al., 2013). It has a close relationship with metabolism and is an important driver in cancer. SRS imaging of deuterated Met has the potential to add to our understanding of these biochemical processes and to provide detailed information at the subcellular level. However, it is important to note that there may be limitations to this approach. For example, it is unclear whether the signal from the deuterium atoms in the methyl group (CD_3_) may be affected by the additional five deuterium atoms present in other parts of d_8_-Met. Additionally, while the CD_3_ group of deuterated methionine provides a strong signal (Hu et al., 2014), it is possible that CD_3_ may have alternative fates aside from methylation. On the other hand, deuterated Met may also be useful for studying other aspects of Met metabolism. A recent study using SRS imaging and deuterated water as a probe found that manipulating the level of L-Met together with insulin can alter *de novo* lipogenesis, the process of synthesizing new lipids, in cancer cells (Fung et al., 2022). This raises interesting questions about the molecular fate under different conditions, such as the role of fatty acid chain elongation and redox control in lipogenesis. Deuterated methionine, along with other deuterated biomolecules, may be well suited to address these questions.

Also, while all our experiments thus far have been carried out on dissected tissue, SRS imaging on tissue *in vivo* has been carried out by other groups but has been limited to accessible tissues or embryos (Wei et al., 2015). SRS imaging does appear to have high potential to record embryonic development in oviparous organisms *in vivo*, should their size permit. Still, the limitation of tissue accessibility for SRS imaging *in vivo* may require miniaturization and an introduction of flexible components or remote technologies. Alternatively, from a systems perspective SRS imaging’s capability to track molecular fate appears to offer high potential for diagnostics using more accessible tissues.

## 5 Conclusion

In this study, we have generated SRS images of d_8_-Met in several dissected *Drosophila* tissues and demonstrated the ability to overcome the challenges that have previously limited long-term uptake of small biomolecules in live cells and tissue at subcellular resolution. In agreement with the known role of mTOR signaling in regulating Met uptake, we provided direct, real-time visualization of mTOR function controlling d_8_-Met uptake and distribution in the fat body. The ability to visualize the range of cell-to-cell differences of d_8_-Met distribution within tissue using SRS has significant potential for basic research on metabolic imaging of less abundant but important amino acids such as methionine and may also have potential for medical applications.

## Data Availability

The raw data supporting the conclusion of this article will be made available by the authors, without undue reservation.
